# Dilemmas in providing resilience-enhancing social services to long-term social assistance clients. A qualitative study of Swedish social workers

**DOI:** 10.1186/1471-2458-12-517

**Published:** 2012-07-12

**Authors:** Anneli Marttila, Eva Johansson, Margaret Whitehead, Bo Burström

**Affiliations:** 1 Division of Social Medicine, Department of Public Health Sciences, Karolinska Institutet, Norrbacka, 3rd floor, 171 76, Stockholm, Sweden; 2 Division of Global Health/IHCAR, Department of Public Health Sciences, Karolinska Institutet, Nobels väg 9, 171 77, Stockholm, Sweden; 3 Nordic School of Public Health, Box 12133, , 402 42, Gothenburg, Sweden; 4 Department of Public Health and Policy, University of Liverpool, Whelan Building, Quadrangle, Brownlow Hill, Liverpool, L69 3GB, United Kingdom

**Keywords:** Social services, Social workers, Social assistance, Qualitative, Resilience, Sweden

## Abstract

**Background:**

Long-term recipients of social assistance face barriers to social and economic inclusion, and have poorer health and more limited opportunities for improving their health than many other groups in the population. During recent decades there have been changes in Swedish social policy, with cutbacks in public benefits and a re-emphasis on means-tested policies. In this context, it is important to investigate the necessary conditions for social workers to offer social assistance and services, as well as the mediating role of social workers between public policies and their clients. Swedish social services aim to promote social inclusion by strengthening the individual´s own resources. We investigated the issues that arise when providing social services to long-term social assistance clients within the framework of resilience, which focuses on the processes leading to positive functioning in adverse conditions.

**Methods:**

Interviews were conducted with 23 social workers in Stockholm and analysed by qualitative content analysis.

**Results:**

The main theme to emerge from the interviews concerned the constraints that the social workers faced in providing social services to social assistance clients. The first subtheme focused on dilemmas in the interaction between social workers and clients resulting from the dual role of exercising authority and supporting and building trust with clients. Working conditions of social workers also played a crucial role. The second subtheme addressed the impact of the societal context, such as labour market opportunities and coordination between authorities.

**Conclusions:**

Overall, we found that social workers to a great extent tried to find individual solutions to structural problems. To provide resilience-enhancing social services to long-term social assistance clients with varying obstacles and needs requires a constructive working environment, supportive societal structures and inter-sectoral cooperation between different authorities.

## Background

People who are socially and economically excluded have poorer health than those who are better off [1-3]. The elimination of poverty has been one of the most basic objectives of the welfare state [4,5]. Benefits for low-income families represent the core of the welfare state and bear an ideological importance: how society caters for the poor is a key indicator of the values of society and the degree of public commitment [5].

Swedish social security and welfare state arrangements result in a comparatively low poverty rate in the population. Most of the public support programs in Sweden are universal and include the entire population. Social assistance, however, is one of the selective and means-tested benefits directed only to those in need [6]. During recent decades, changes have been introduced in Swedish social policy, including cutbacks in public benefits, tighter eligibility criteria and a re-emphasis on means-tested policies [7,8]. Poverty has increased among households receiving means-tested benefits in Sweden (as well as in the other Nordic countries) [8]. In this changing situation it is important to investigate the conditions necessary for the effective conduct of social work [7], and the role of social workers as mediators between public policies and their clients [9].

### Social assistance recipiency

In 2010, 4.7% of the Swedish population received social assistance on at least one occasion during the year. About one third of them (37 per cent) were long-term recipients (social assistance for at least 10 months). [10] A large proportion of recipients of social assistance had a refugee or immigrant background [11]. Young adults, lone mothers, and single men and women were other common groups [12]. There was considerable variation between residential areas in the proportion of people who received social assistance [10].

The number of people on social assistance declined after the 1990s, while its duration increased: those on social assistance received it for longer periods [13]. Reasons for social assistance receipt have been put forward at both the structural and individual level. Weak ties to the labour market, low educational level, poor political and financial resources, refugee status and poor health are common characteristics among long-term social assistance recipients [14]. Those with fewer resources and more problems tend to stay on assistance, and the longer the period of recipiency, the more difficult it becomes to exit [15].

### Social assistance as part of social services

Social assistance in Sweden is part of broader social services and is governed by the objectives and commitments of the Social Services Act of 1982, a goal-oriented framework law [16]. The objectives of the Act are to promote economic and social security, equality and active participation in society. According to the Act, social services should support individuals “*to release and strengthen their own resources”* and be based on “*respect for the individual’s self-determination and integrity”* (p. 64), with the aim of promoting social inclusion [17].

The provision of social services, including social assistance, in Sweden is the responsibility of municipalities, which have the autonomy to choose the organisational form of their activities. This may result in differences between municipalities and individual social workers in how they interpret the Act and provide social assistance [18]. A national benefit standard was introduced in 1998, obliging all municipalities to provide a minimum standard of assistance [10]. Social assistance claimants must comply with the requirements laid down by social services, such as actively seeking jobs, studying Swedish language or participating in job-seeking activities. If the claimant does not comply, financial sanctions can be used [19].

### Social workers as mediators between public policies and clients

Front-line professionals, like social workers, interpret and fulfil the ambitions of government policies and channel different services to the citizens [9,20,21]. Swedish social workers have a relatively broad discretion [9] in their work, but have to follow political decisions [22]. The needs and desires of the clients are transformed in a process to fit the organization’s administrative categories [21].

In this context, Lipsky´s work [23] on the mediating role of ‘street-level bureaucrats’ between the public institutions and the clients is still highly relevant. Debates in the current social work literature, especially in relation to managerialism, argue that policies have been implemented in Western societies to regulate social welfare and other public services and to gain control over professional practices. Whether the discretion of social workers continues to operate or has been curtailed by these policy shifts is a matter of debate [20,24].

It is clear from the literature that social workers are in a position of power in relation to their clients. According to Järvinen (2002), social work has always been a form of help rather than a service [25]. A help relation is linked with symbolic, “invisible” power that lies hidden in kindness, help and bringing up, which has been identified as a legitimate role [25,26]. In social work, distinctions are sometimes made between deserving and undeserving clients, easy cases and difficult cases [22,25].

### Social services as resilience enhancing?

The intentions of the Social Services Act resonate to a great degree with the concept of resilience and its focus on positive outcomes in adverse conditions [27-31]. Much of the resilience literature relates to risks and protective factors in individuals´ living conditions, less is known about the processes under stress and how risks and protective factors accumulate over time [32]. Resilience research, with its focus on health and strengths of the individuals and communities, can contribute to knowledge about how to change the odds under adverse circumstances, which in turn may be transferred into programs and policies when appropriate [27,32,33].

The importance of the social-environmental context in positive development has been acknowledged in resilience literature. That is, that resilience is not a condition of individuals alone, but related to the vulnerability and protective factors in an individual’s environment [27,28]. Seccombe (2002) further argues [34]: “*Resiliency cannot be understood or improved in significant ways by merely focusing on these individual level factors. Instead careful attention must be paid to the structural deficiencies in our society and to social policies that families need in order to become stronger, more competent, and better functioning in adverse situations”* (p 385). The importance of creating settings in which individuals have opportunities to grow and develop, despite the risks, has been recognised also in the ‘health assets’ literature [33].

In this study, the term ‘resilience-enhancing services’ refers to processes in social services aimed at strengthening clients´ own resources and capabilities, based on respect for their integrity and self-determination, and thereby strengthening their functioning in adversity.

Is it possible to develop social services including social assistance to be more resilience enhancing? What are the dilemmas involved? In previous studies, we investigated the experiences of living on social assistance from the perspective of recipients [35,36]. In the present study, we focused on the social workers who provide services. In the discussion section we reflect on our findings from the resilience perspective. The aim of this study was to investigate issues that arise when trying to provide resilience enhancing social services to long-term social assistance clients.

## Methods

### Setting

Our primary purpose was to explore experiences of social workers providing services to long-term social assistance clients in different socio-economic areas and with different population groups to enable us to compare and contrast their experiences [37]. We conducted interviews with social workers in six sites in Stockholm County, providing a variety of geographical settings with differing socioeconomic compositions. Three areas were multicultural, one ‘mixed’ area including neighbourhoods with low and high socioeconomic status, one outer ‘working class’ area characterised by relatively low educational level, and one affluent area where the number of households receiving social assistance was low, but where many recipients had received the benefit for a long time.

The number of households receiving social assistance in the studied areas varied at the time of the interviews from approximately 240 to 1200 depending on the size and socioeconomic composition of the areas (low number in the outer and affluent area, and high number in socioeconomic disadvantaged areas and in a mixed, geographically large area). The caseload varied within areas and between different social work teams. Those working with young adults usually had 25–30 clients per social worker, whereas those working with older adults in the same areas or in different teams had an average caseload of between 60 and 80 households per social worker.

During the economic recession in the 1990s, the number of households receiving social assistance increased dramatically in Sweden, and started to decline at the end of the 1990s, but slowly among long-term recipients [14], also in the areas included in this study^a^.

### Sample

A purposive sampling strategy was employed, to generate a sample suited to the specific needs of the study [38]. Through the social assistance manager in each area we came in contact with social workers with experience of working with different groups within social assistance (unemployed, refugees, young adults, persons with chronic illness and drug abusers). The first author (AM) conducted 23 interviews with social workers (three to five interviews in each of six study sites). Five additional interviews were conducted with professionals working with persons and families in disadvantaged conditions, but not necessarily with social assistance recipients. These five interviews were excluded from the analysis in this study. The sample consists therefore of 23 interviews.

All 23 interviewees were social workers; three of them worked with activities directed to social assistance clients, the other 20 with administration of social assistance. Six worked as managers. Seven interviewees had at most two years in their profession, five from three to nine years, and 11 had more than ten years. About a third of them had an immigrant background. Two interviewees were men, which reflected the gender balance among social workers in the field in the study areas.

### Data collection

Interviews were conducted in 2005–2006, with each interviewee on one occasion by the first author. All interviews were tape-recorded (with the interviewees’ permission). After each interview, short notes were taken about the content of the interview and other issues that emerged during the interview. Before the interviews were carried out, the first author gathered data from the managers of the social assistance units in each of the study sites. Managers gave information about the caseload, the current development in their area, and the organisational structure and changes. These discussions were documented in field notes, together with notes taken after each interview.

In addition, in one of the studied areas, the first author performed observations of social workers´ overall activities, documented in field notes. The aim of the observations was to gain better understanding of the organisational context in which social workers acted. The field notes served mainly as a source of information about the context of social assistance work in the study areas. Field notes were in line with the current literature on the development of the social assistance in Sweden and influenced how we framed the study as a whole. During the analysis process, we repeatedly came back to the field notes, to reflect on our emerging categorisation, and to check whether there were issues which were not taken into account in the categorisation.

The interviews lasted from about 1 to 1.5 hours. Interviewees were informed about the aim of the study and their right to withdraw, and that their responses would be kept anonymous. An interview guide was used, which included open-ended questions about the services the interviewees provided, the clients they met, their role and experiences of their work. Interviewees were asked about the needs of the clients, goals of their work and why the clients were applying for social assistance. Interviewees were not asked directly about resilience; they were asked to reflect on what they perceived as “good” social services and obstacles in providing such services. The interviews took place at social welfare offices or in centres to which clients were referred. According to the Regional Ethical Board in Stockholm, section 5, the study did not require ethical approval under local guidelines (dnr 04-609/5).

### Analysis

All interviews were transcribed verbatim. The transcripts were read several times to obtain a good overview of the material. Subsequently, open coding was performed line by line by the first author. After that, a number of content areas [39] were identified in discussion with all the authors. We selected for this present study the content area “values and norms” for further analysis. All sequences within this content area were brought together into one document. The selected content area dealt with sequences in the interviews concerning traditions and values guiding the social work practice and sequences where values among clients were discussed. Different views were described between social workers and clients regarding, for example, the activities to which clients were referred, and frustration among social workers linked to the organisation in which they worked.

Subsequently, the combined document was read through several times. Data were analysed using qualitative content analysis in line with Graneheim and Lundman (2004), an appropriate method in the analysis of personal experiences [39]. After the open coding, the categories, subcategories and main theme were identified to capture the underlying meaning of the text.

The research team, consisting of all the four authors, contributed continuously during the analysis process. Discussions in the team arose, for example, about the labelling of the subthemes and possible overlapping categories. In the analysis we tried to capture the variation in the material (including discrepant data). Constant comparison [37] characterised the analysis process, aiming to find differences and similarities in the data.

In the presentation of the findings the names of the interviewees have been changed to protect interviewees´ identity. Two of the interviewees were men. When we use quotations from them, we have used female names to protect their identity. Quotations were selected to illustrate the issues which emerged from the data.

## Results

The main theme emerging from the interviews concerned constraints in the provision of social services to long-term social assistance clients. Two sub-themes related to interaction with clients, and the societal context in which they operated. See Table 1.

**Table 1:** Main theme, subthemes and categories

**THEME**	**Constraints in the provision of social services to long–term social assistance clients**					
**Subthemes**	**Dilemmas in the interaction between social workers and clients**				**Societal influences on the interaction between social workers and clients**	
**Categories**	Balancing the dual role	Obligations vs. rights of the clients	Clients recognized as individuals vs. seen as categories	Building trust	Authorities conflicting views and expectations on clients	Labour market opportunities and overall policy context

The first subtheme included four categories: balancing the dual role, obligations vs. rights of the clients*,* recognising clients as individuals vs. groups, and building trust with clients. The second subtheme consisted of two categories: authorities´ conflicting views and expectations of clients, and the labour market opportunities and policy context. Categories were sometimes interwoven in the interviews. The following sections present the findings in terms of the themes and categories depicted in Table 1.

### Main theme: Constraints in the provision of social services to long-term social assistance clients

The way that the interviewees perceived their work and their possibilities to offer support influenced the interaction with their clients. Specific challenges were highlighted which related to interviewees’ possibilities to work actively with each client, often in multicultural contexts. The status of their work and overall societal structures were other prominent aspects discussed in the interviews.

Unemployment was mentioned as a major problem and reason for clients applying for social assistance. The social workers distinguished clients who were ”*fit to work*” from those, who had additional needs, like addiction problems and/or health problems and who were “*far away from the labour market*” and needed other solutions, taking more time. One way to handle the number of clients was to concentrate on the clients closest to the labour market, to “manage them off” social assistance in various ways.

Two of the study areas had an overall reputation in social services of being innovative and oriented towards development, which interviewees from these areas mentioned as something positive. Active leadership with visions and goals, stability in working groups, emphasis on working methods and support in working groups were aspects mentioned as essential requirements to “*reach results*” in the work with social assistance (combined with a good labour market situation). Most of the interviewees talked about the importance of “*working actively with clients*”.

"“You need resources if you work actively, resources to employ social workers…but in the end it gives returns, that is, you save money. But this work is not only about financial resources and how many social workers there are… You also need a conscious strategy of how to work methodically. The content is of major importance.” (Lisa)"

Especially in multicultural areas with heterogeneous ethnic backgrounds among clients, it was said to be “*exciting*” and ”*interesting*” to work in these areas, where “*the needs were huge*”. Differing views concerning labour market participation among men and women and perceptions of illness and health were reflected. The interviewees in these areas expressed extra pressure to give information about the social welfare system and rules regulating social assistance. Many clients were also newly arrived in Sweden and had limited knowledge of the Swedish language and welfare system. Providing clear information was therefore mentioned as a significant part of the social workers´ task.

Social assistance work was described as a less attractive field in the social work arena. Interviewees who were relatively new in their profession pointed out that they were not going to work with social assistance for long. Generally, the work was perceived as quite stressful, especially at the end of the month, but at the same time “*never boring*”. New things happened all the time and they constantly had to deal with clients´ different kinds of “*acute crisis*”.

Social workers explained that in their work they faced structural problems such as lack of jobs for clients with reduced working capacity because of ill-health, and for clients with a short educational or an immigrant background, especially with limited knowledge of the Swedish language. It was a complicated task to try to find solutions for groups and individuals, when the opportunities and possibilities were limited by the societal context, and when the goals and expectations among different actors differed. Their possibilities as social workers to solve these kinds of problems were limited.

"“…in Sweden we have politics aiming at providing equal opportunities for all people but at the same time there is a directive to be restrictive and careful when granting for example sickness benefits…There is a double message in this…How can we have an ideology emphasising equal worth of all people and equal participation in society, but at the same time restricting…people´s return to the labour market?” (Olivia)"

### Sub-theme one: Dilemmas in the interaction between social workers and clients

Social workers both supported and made demands on their clients to do specific tasks. How they managed to handle this dual role, and to treat clients as individuals, influenced the trust between social workers and clients, which was described as essential in work with long-term clients in particular.

#### Balancing the dual role

In interview accounts we found the dual roles of social workers as a recurrent concern: on the one hand social workers demanded and expected clients to do certain things and on the other hand had to act as support, help and guide for them. The demands made by social services included the requirement that clients actively seek jobs and participate in training and other activities to which they were referred. The helping part of the work consisted of trying to find solutions to clients´ problems as well as building trust in the client-professional relationship. It was sometimes problematic to combine these two roles, as Helen expressed:

"“We work with exercising authority and it includes both support and help but also some suspicion and control. Suspicion and control are the tough parts, but we have to find a balance there.”"

How social workers shared their time between exercising authority and supporting clients varied. Social workers stated that they work under the legal framework and follow the rules of their organization, but they still had some discretion to decide how to work at “micro level”.

"“I feel I can control pretty much myself … If I choose a client who I want to work with in a special methodical way … then maybe I choose this client now this month … and then I take someone else in another month…I have never been able to work with them all at once.” (Sonja)"

A focus on assessment and administration related to monthly payments was an essential part of the work and in many situations, especially when the caseload was heavy, this dominated the helping and supporting part of the work. Some social workers talked about their desire to do “*real social work*”, by which they meant supporting people to change their lives.

Interviewees with long experience talked about the importance of accepting the dual role and even using the role of an authority as an instrument to “push their clients to change”. Some, who were new in their profession, on the contrary, reported that they had to follow and act according to the rules and regulations, sometimes against their own convictions. The need for negotiation between clients and social workers was obvious, especially when the goals differed between the two. This was illustrated, for example, by clients´ participation in activities that social workers referred them to. Many cases were described where the clients did not see the activities as meaningful.

As a public official, the social worker had to make decisions about “acceptable” solutions in line with the organisational rules, regulations and practice, and inform the client about the rules and other possible solutions to support themselves. They had power in relation to the clients, which several social workers suggested should be more reflected in their working organisations:

"“You have extensive power as an authority towards clients. You make decisions…you have access to allocating money. We have to be aware of this role…and understand what power we have over people…to be humble in interaction with the clients.” (Lisa)"

#### Obligations vs. rights of the clients

Obligations and rights of the clients were discussed in the interviews with emphasis on clients´ obligations as social assistance recipients. Some clients were perceived to demand their rights, but did not recognise their own obligations. During recent years, with economic constraints, the control and demand on clients had become more central in social assistance. One interviewee explained:

"“There are those clients who think social assistance is a right without obligations. One cannot just receive money because they do not have any…it is not that simple. It has always been so, but it was much easier before. Now there is more control. We have to do a lot of paperwork…and we have to find out how clients live, with whom they live…tax papers, bank statements, and transactions…”(Irma)"

Expectations from social services towards clients were discussed as something positive in the long run for the clients. The provision of money alone was not seen as a long-lasting solution. According to most interviewees, when social workers expected and demanded that clients do things, and encouraged their clients’ belief in their own capacity to solve problems, this could in a long run help clients out of benefit recipiency into independent life.

"“…you have to place the responsibility on the individual and you have to make demands. You have to bring out the strength of the individual…I believe that if you make demands on people, they usually also find solutions to their situation.” (Ulla)"

#### Clients recognised as individuals vs. seen as categories

Clients were described as a heterogeneous group of people with differing needs and obstacles. The attitudes of social workers towards different clients and client groups influenced the way they described their interaction with their clients and also partly formed the expectations they had of them. Involvement of clients in the planning of their own cases was discussed as something that should be developed.

"“…it is the social worker who writes the action plan or planning … when we as an authority suggest something, the client agrees…This is a really big area to develop, to include small steps, not these huge jumps forward but small steps, which the client agrees to. And this should be discussed and revised during every visit.” (Helen)"

The social workers perceived that it was important for clients to be treated as an individual. With a high caseload, however, there was little time to meet clients and get to know them as individuals, and they were referred routinely to certain activities. During the economic recession of the 1990s, the average social worker caseload was described as high, especially in some of the study areas. One interviewee compared her work during and after recession:

"“Last fall, I had 139 cases. But now when I have 50 … now I see that I could not have done a good job … it became of course emergency-driven. … Right now…I know who the clients are, I know where they are, in which phases … If they are going to move forward I must also be able to meet them frequently.” (Rebecca)"

Interviewees had different attitudes to certain client groups, with more sympathy for some than others. In most areas, social workers had the opportunity in their working teams to reflect on their reactions to different clients and how to handle these feelings. In general, it was perceived as easier for a single person than for families with children to live on social assistance. Overall, interviewees felt strongly for children, and many discussed the negative effects for children of living long term on social assistance, like poor self-esteem and feeling as a burden to their parents. Interviewees reflected on how it was for children to live in families with low income for a long time.

"“…children see that the other children have bicycles or skates. You perhaps never get new skates, but used ones. Children I think are quite aware of these kinds of things…My children can also get used ones, but they know that we can afford to buy new ones if we want to. We have made a choice how to use our money. …These families cannot afford this little extra, they live on the margin all the time. They have no possibility to choose.” (Ylva)"

Young adults, with difficulties to enter the labour market, were identified as the most important group to prioritise and work with. The common statement among the interviewees was: “*young adults should not start their adult life on social assistance*”. They were also prioritised; the aim was to handle them quickly in order to prevent passivity. In several interviews, young adults were compared favourably to older persons with addiction problems, who were seen as less deserving.

In the interviews, social workers mentioned that it was “*easier*” to work with clients who were motivated, willing to develop, to find solutions in their life situation and to accept the prevailing normative values, like the notion that both men and women should work to support themselves. Clients perceived as “*unmotivated*” or as not willing to share the goals of social workers and social services were seen as difficult. Interviewees also described frustration, when they talked about people who tried to cheat or applied for social assistance when not entitled to it. In these cases, social workers felt that they had spent their time and energy “*for nothing*” while clients “*who really are in need*” got less attention.

#### Building trust

Many clients received social assistance for a long period of time. Social workers therefore found it important to create a relationship, but building trust with clients took time. The disadvantaged position, and in many cases different cultural backgrounds of the clients, added to the clients´ suspicion and was not conducive to trust.

"“A person, who comes here is suspicious… what is this about… you meet an authority. As an authority you influence people’s lives with money. But if you start to inform in a good way and explain our role and make it clear that we are here to help…then suspicion can change into trust that your social worker doesn’t work against you, but with you.” (Karin)"

In the interviews, expectations of clients towards social services and social workers were reflected. Many clients were immigrants and refugees from other parts of the world, and some of them had limited knowledge about the Swedish social welfare system. Social workers reported that clients’ expectations of Swedish social services were often high:

"“… they cannot see the difference between social services, social insurance office, child allowance or whatever… and they have hopes and expectations that we can solve everything…: we can fix apartments, we can fix day care for children tomorrow…But we don´t have these possibilities. And we feel a huge frustration and disappointment directed towards us…” (Ingrid)"

Interviewees also discussed difficulties in building trust and making progress with clients who were ‘hard to reach’ and had difficult life experiences behind them, often combined with years of contact with social services. The importance of not losing hope was emphasised.

### Sub-theme two: Societal influences on the interaction between social workers and clients

The societal context forms a framework in which social workers operate. For most clients the receipt of social assistance required collaboration between different agencies like health care services, the social insurance office and unemployment office. Exit from social assistance might require medical rehabilitation, work practice or medical certification for disability pension. The differing views and expectations on clients among the various agencies, as well as the overall construction of the welfare system, were considered of great importance.

#### Authorities conflicting views and expectations of clients

Different demands by different agencies, or conflicts between them in their views on the clients, were common. Social workers described how clients often had to balance between the differing demands. A joint plan between the various public agencies was lacking concerning the clients seeking support from different authorities.

Several interviewees stressed that those working with social assistance were seen as “*the bad guys*” by other public agencies, who had difficulty in understanding the rules and demands they as administrators of social assistance had towards their clients, as well as social workers´ emphasis on putting responsibility on clients, instead of helping them “*too much*”.

"“It upsets me, when the other actors involved are calling and nagging… they question our decisions and judgments. It is very, very frustrating… that you put so much energy into explaining, not only to the client … but also to other professionals, who do not behave professionally… They call and say we have to grant social assistance.” (Ulla)"

Cooperation between different units within social services, as well as with other actors like health care providers, was part of the daily social assistance work. Situations where cooperation was lacking were described, and where the goals between different agencies towards their clients differed. More contact with the various services involved and understanding of their different ways of working with the same clients was needed.

"“…we need a better contact with social insurance office and unemployment office for example. Meet each other. To sit and discuss…how we think about different things. That we could agree on a joint way to think, and increase the understanding for each other’s work… (Emilia)"

#### Labour market opportunities and overall policy context

Social assistance is the ultimate safety net in the Swedish society. Hence, people in need of financial (and other support), who are not entitled to social insurance or unemployment benefits resort to seeking social assistance. How the society and welfare state institutions function in general has a great impact on which clients seek social assistance and the needs the clients have. Birgitta who worked mainly with immigrants and refugees described the heterogeneity among her clients:

"“All clients are different. We have unemployed with mental or physical disabilities who are not covered by social insurance. We have those who are fit to work but who don´t know Swedish language. Then we have those, who are seeking jobs, but have not been successful."

Interviewees concluded that to a great extent, especially in disadvantaged areas with high unemployment and many refugees, they work with structural problems realised at individual and group level. One example was the lack of opportunities on the labour market for people with reduced working capacity. Much more effort was called for, not least in areas with many clients with differing backgrounds, health problems and multiple needs:

"“… You need more options. Some older men, for example. Their feeling is that they are retired although they may be in their 50s…we need alternative employment without significant demands on either Swedish language or strict attendance, but participation instead … They would never consider to work because they are so sick, but there is no medical certificate showing that. (Irma)"

Interviewees also considered that there was still much to develop in terms of Swedish language training for immigrants and ways to enter and remain on the labour market, especially for the majority of clients, who have few years of schooling. For many clients it took years to find a job and to learn the language. Prompt efforts from social and welfare services to the clients were seen as crucial.

Interviewees found it difficult to solve the income problems of clients with low education, often combined with other obstacles. A feeling of “constant failure” in life was described among many clients; clients tried to solve their situation without success. The goal in social assistance work according to all interviewees was to assist clients to support themselves in other ways than social assistance. Their task was to use their knowledge and effort to strengthen the individuals to find alternative solutions. In those cases, where it was not possible, they aimed to work for better quality of life among clients with the final aim of minimising social exclusion.

"“They [clients] should become a part of the society. Often they are a bit outside, they are falling out somehow, they are isolated…” (Ellen)"

## Discussion

This study identified a number of dilemmas encountered by social workers in their efforts to provide resilience-enhancing social services to long-term social assistance clients. We discuss our findings from the resilience perspective based on the aims of the Social Service Act. Two main dilemmas were found; the first one concerned social workers in the interaction with their clients and the second one the societal influence on this interaction. Three aspects of these dilemmas are particularly pertinent and their implications are discussed below.

### Managing the dual role

A fundamental difficulty in the work with social assistance concerns balancing the dual role between supporting clients and exercising authority. The social service agency is by its nature administrative and controlling [22,40,41]. Legislation and rules as well as social workers’ personal preferences and experiences determine whether the individual gains access to services and support. In this study, social workers who were new in their profession perceived the dual role as especially difficult to handle. As in previous studies [42], our interviewees emphasised that quite often the goals and priorities of clients and social workers differed. The interaction between the social worker and the client consists of negotiations in which the power is unequal [22,25]. Hermodsson (1998) found that clients’ possibilities to influence the decisions vary between different client groups, and that the most disadvantaged have most difficulty to defend their civil rights [42].

In this unbalanced context, it may be difficult to provide resilience-enhancing social services, trying to balance between supporting clients’ preferences, and making demands on them to believe in their own capability, to make them move beyond what they perceive as their own limitations. Many clients had also been in contact with social services for years. The challenge for service providers is to recognise the small steps forward in the daily life as a success to build on [36,43] and reflect over the “hidden” resilience among clients [43,44]. Overall, building trust with clients was said to take time, but it was necessary for strengthening clients, especially with clients with difficult life experiences and often a feeling of constant failure. Building trust was an example of potential protective processes [45] facilitating successful functioning.

Based on the findings of this study, one key element in enhancing resilience of clients includes making them active participants in decisions made about them. It was stated that an individual action plan for each client, to be revised continuously based on the client´s situation, obstacles and capacities, would facilitate this process. In practice social workers, as other street level bureaucrats [9], have to balance the need to recognise clients as individuals and working with different groups with similar kinds of need.

### The organisation of social services

Interviewees expressed that the organisation and working conditions in social services influenced their possibilities to support their clients. Categorisation of clients in social services into groups [9,22] is common, for example in our study between those who were and were not fit for work, those who were “easier” to help and “more demanding or difficult groups”.

The need for social workers to recognise and reflect on their discretionary power and differing attitudes towards different client groups was discussed as one key element in their professional role. Interviewees wished to work “*actively*” and “*socially*” with each individual client to strengthen the client in the long run. According to the interviewees, that was what they as “*social workers should do*”. In line with other studies, this study shows that the discretionary power of social workers [9] today is still under the influence of managerialism, and is a relevant issue to study further [20,24].

A manageable caseload, an active leadership with visions and goals, reflection in working teams and systematic way of working methodically were mentioned as aspects of good and stimulating work environments. The opposite: a heavy caseload, a feeling of never managing the day, no support from working team or managers and the instability in working teams were mentioned as obstacles and taking time from the supporting part of the work. This is in line with the findings by Tham (2007) and Tham and Meagher (2009) who studied working conditions of social workers (especially in child welfare) in Stockholm County [46,47].

### The prevailing conditions in the surrounding society

Social exclusion has been seen as a major risk for groups living on welfare benefits for a long time. In Sweden, one welfare policy aim has been the inclusion of the groups at the margins of society [14]. The construction of the welfare benefit system, combined with labour market opportunities, have an important impact on how successful the social services are in moving clients out of social assistance recipiency and supporting resilience among the clients.

Social assistance is meant to be a temporary solution but the long-term receipt of the benefit has increased [13,19]. This worried interviewees in our study especially when it came to families with children, regarding the possible consequences of long-term receipt of social assistance on children. Alternative labour market opportunities and possibilities for immigrants and young adults to enter the labour market were needed, especially for persons with reduced working capacity. Societal efforts to improve the living conditions of these groups as well as ensuring a more inclusive labour market are examples of potentially important supportive structures (see for example Seccombe, 2002 [34]) which would facilitate the practice of social work.

Interviewees described how different authorities often worked separately with their clients, with disparate demands and views on what would be the best for the client. A joint discussion among the actors together with the client concerning views, demands and goals and the client´s position between them, would facilitate strengthening clients in adverse conditions. Sweden has a national strategy for social inclusion [48] but further efforts are needed according to this study to prevent social exclusion among the disadvantaged groups who depend on social assistance for their living or fall between the stools between social assistance, unemployment benefit and sickness insurance administered at different levels in the welfare system.

## Conclusion

Overall, we found that social workers in our study were, to a great extent, trying to find individual solutions to structural problems. To provide resilience-enhancing social services to long-term social assistance clients requires a constructive working environment, supporting societal structures and inter-sectoral cooperation between different authorities. Further studies are needed concerning how to develop clients´ possibilities for self-determination and integrity as social assistance clients, and how public institutions can support the provision of resilience-enhancing social services.

## Endnotes

^a^ The process of becoming a social assistance client followed a set procedure in the study areas. People who wished to apply for social assistance were referred to the intake team in social services in the local community where they were living. In the intake team, social workers investigated if the clients were entitled to social assistance. The intake team took care of clients who had temporary needs (usually less than two-three months) for financial support. After that, the client was usually referred to other, specialised working teams within the social assistance unit.

## Competing interests

The funding organisations had no role in the study design, data collection, analysis, interpretation or writing this article. The authors declare that they have no competing interests.

## Authors’ contributions

AM participated in the design of the study, carried out the interviews, in the analysis of the data and drafted the manuscript. EJ participated in the analysis of the data and preparing the manuscript. MW participated in the design of the study, in the analysis and preparing the manuscript. BB participated in the design of the study, in the analysis and preparing the manuscript. All authors have read, revised and approved the final version.

## Pre-publication history

The pre-publication history for this paper can be accessed here:

http://www.biomedcentral.com/1471-2458/12/517/prepub
